# Physiological responses of three field-grown species (*Ceratonia siliqua*, *Eucalyptus camaldulensis,* and *Moringa oleifera*) to water deficits in a Mediterranean semi-arid climate

**DOI:** 10.1038/s41598-023-31664-y

**Published:** 2023-03-20

**Authors:** Hasna Ezzine, Mohamed Louay Metougui, Hassan Boukcim, Younes Abbas

**Affiliations:** 1 Polyvalent Team in Research and Development, Polydisciplinary Faculty, Sultan Moulay Slimane University, Beni Mellal, Morocco; 2 AgroBioSciences Department, Mohammed VI Polytechnic University, 43150 Ben Guerir, Morocco

**Keywords:** Physiology, Plant sciences, Plant physiology

## Abstract

Reforestation of degraded drylands calls for the selection of species with the capacity to withstand water scarcity. In this current study we have assessed, the physiological responses of three field-grown species (*Ceratonia siliqua*, *Eucalyptus camaldulensis* and *Moringa oleifera*) to water deficits in semi-arid regions in order to suggest a potential species for rehabilitation programs. The physiological behavior of the given species was studied in three irrigation schemes: subsurface drip irrigation (applied weekly), tank irrigation (applied monthly), and unirrigated plants. In a stressed state, an assessment of relative water content (RWC), water potential (pre-dawn water potential PWP and midday water potential MWP) and stomatal conductance revealed three contrasting physiological responses. First, *C. siliqua* stomata remained open with a high RWC at low water potentials. Consequently, this species tolerated water deficits by decreasing its leaf water potential, primarily associated with osmotic adjustment. On the other hand, *E. camaldulensis* was found to be a drought-avoider species, mutated to a water-saving strategy by complete stomatal closure. Finally, for the extreme case, *M. oleifera* showed leaf shedding under water deficit conditions. These different physiological responses allowed these species to survive water deficits, and consequently, could be considered suitable candidates for rehabilitating degraded semi-arid areas.

## Introduction

Arid and semi-arid regions cover a third of the world's continental areas^1^. These regions are vital to the global environment^2^. Despite their importance, drylands are continuously degraded by a complex combination of climatic and human-induced stressors, such as rising temperatures, low mean annual rainfall, high water evaporation, overgrazing, and unsustainable agricultural practices^3^.

Dryland land degradation, also called desertification, has affected Africa, Asia and Mediterranean Europe for centuries, the Americas for the last 200 years, and Australia over the last century^4^. The main ecosystem degradation processes include vegetation degradation in rangelands, tree cutting, water and wind erosion, and salinization of irrigated lands^4^. Africa is widely held to be the worst affected. In 2002, the United Nations Environment Programme (UNEP) claimed that 45% of the continent was seriously desertified^5^ and 73% of its agricultural lands were degraded^6^^.^ Morocco is not exempt from this crisis, as desertification affecting large areas is more pronounced due to the arid climate and soil vulnerability to erosion. Moreover, the precariousness of rural populations pushes them to overexploit natural resources to satisfy their increasing needs, worsening the deterioration of their surroundings^7^. Several solutions have therefore been adopted to rehabilitate degraded lands. The main biophysical solutions are grazing management, conservation agriculture, land-use change, hedge barriers, windbreaks and revegetation, leading to better rainfall infiltration, more soil moisture, and improved soil organic carbon levels^5,8^.

The revegetation of arid and semi-arid areas is a suitable measure against drought, which influences negatively the seedling stage in rehabilitation programs^9^. It induces a diverse set of morphological, anatomical, physiological, biochemical, and molecular responses in plants that provides the ability to cope with water stress conditions depending on the intensity and periods of stress, the developmental stage of the plant, and the genotype itself^10^. In response to drought, plants typically reduce their leaf size, shoot growth and stem diameter^11^. Moreover, developing an efficient root system is another morphological strategy to avoid water stress^12^^.^ In addition, plant tissues exposed to drought generally exhibit a small cell size and a large vascular tissue with a thicker cell wall^13^. Drought also decreases stomatal conductance and leaf water potential^13^^.^ Plant species are classed as isohydric or anisohydric, depending on their stomatal behavior. Isohydric plants close their stomata quickly, limiting water loss and carbon dioxide uptake^14^. In contrast, anisohydric plants delay stomatal closure and maintain photosynthetic activity^14^. These plants are drought-tolerant and are able to maintain cell turgor by accumulating various osmolytes, improving water uptake^15^. At biochemical level, drought triggers the production of reactive oxygen species (ROS), such as H_2_O_2_, O_2_^-^, and OH radicals in plant tissues, resulting in membrane lipid peroxidation. In response to this damage, plants synthesize many antioxidant enzymes, which collaborate to maintain the integrity of plants^16^.

Furthermore, drought induces the expression of numerous genes encoding the enzymes and proteins involved in plant responses to drought^15^. The ability of plants to survive drought is defined as drought tolerance^17^, which is a complex trait that impinges on several mechanisms referred to as: (i) escape, through acceleration of the plant reproductive phase before water stress that could hinder its survival, (ii) avoidance displayed by an endurance with increased internal water content as a response to water loss and enhancing water absorption^18^ (iii) tolerance expressed by an endurance with low internal water content by maintaining cell turgor through the improvement of osmotic adjustment^19^.

Rehabilitating degraded semi-arid and arid regions under current climate change requires the selection of drought-tolerant species. Studying the water status of plants under field conditions using a physiological approach will therefore provide sufficient insights into their behavior in response to water deficits. With evidence of global warming and rapidly changing environments, plant species that are locally adapted risk being unable to cope effectively with the new conditions^20^. Due to this fate, combining native and exotic species in rehabilitation programs is a new tool for testing a plant's plasticity to tolerate new environmental conditions.

In this study, we focused on the physiological behavior of three species: *Eucalyptus camaldulensis, Ceratonia siliqua,* and *Moringa oleifera.*

*Ceratonia siliqua* is an evergreen species successfully established in the Mediterranean climate with a long dry season. Previous studies have reported that carob trees avoid drought by establishing a deep root system and wide vascular tissues^21^. Additionally, carob trees can withstand drought by closing their stomata and keeping a high relative water content during drought seasons^22^. However, Gadoun et al. (2019)^23^ showed that carob seedlings growing under controlled conditions react to drought by accumulating certain osmolytes, such as carotenoids, chlorophyll a and b, and proline.

*Eucalyptus camaldulensis* is an evergreen species widely planted around the world. It is well known for its industrial wood benefits^24^. Eucalyptus plants exhibit different mechanisms in response to drought that have been summarized as transpiration limitation by controlling stomatal opening^25,26^, extension of the root system to gain access to water in deeper soil layers^27^, and lowering of the plant's water potential through the accumulation of cell osmolytes^28^.

*Moringa oleifera* is a fast-growing medicinal species native to India and cultivated in semi-arid and arid regions worldwide^29^. Unlike carob and eucalyptus plants, drought has been found to not significantly affect stomatal conductance in *Moringa oleifera* seedlings^30^^.^ In contrast, this species was found to overcome drought by synthesizing a wide range of secondary metabolites (phenolic compounds, flavonoids, and proanthocyanin)^31^, as well as mineral compounds (nitrogen, phosphorus, potassium, calcium and magnesium)^32^.

This study sought to assess the physiological behavior in a semi-arid region of three field-grown species (*Eucalyptus camaldulensis*, *Ceratonia siliqua,* and *Moringa oleifera*) belonging to three different families (Myrtaceae, Fabaceae, and Moringaceae) in response to water deficits. The ultimate goal was to suggest suitable species for rehabilitating degraded arid zones.

## Material and methods

### Study site 

This study was carried out in spring 2021 on the experimental farm belonging to the Mohammed VI Polytechnic University (32° 13′ 6.67″ N, 7° 53′ 14.44″ W, 450 m.a.s.l) in Benguerir, Morocco.

The region is characterized by a hot semi-arid climate with a mean annual evapotranspiration (ET0) of 1600 mm. Over the last ten years, the average day temperature has ranged between 18 °C in winter and 37 °C in summer, while the average night temperature has ranged between 9 °C in winter and 22 °C in summer. The annual average rainfall was between 150 and 250 mm, with 65% mostly distributed between October and March.

The experimental soil at the site was loamy to clay loamy shallow calcisol, 60 to 80 cm deep with a petrocalcic horizon, alkaline (pH 8.7), having low electrical conductivity (0.15 dS m^−1^) and an average organic matter content of 1.7%.

### Plant material and experimental design

The studied species were *Ceratonia siliqua*, *Eucalyptus camaldulensis,* and *Moringa oleifera,* cultivated on the experimental farm belonging to the Mohammed VI Polytechnic University as part of the “Carbon farming and Afforestation of Arid Zones” project. These three forest species are not listed as endangered species, or species at risk of extinction, according to the IUCN Policy Statement and the Convention on the Trade in Endangered Species. The National Agency for Water and Forests of Morocco provided the carob and eucalyptus seedlings. The seedlings were produced following Moroccan seedling production quality standards before being planted at the reforestation site. On the other hand, *Moringa oleifera* seeds were bought from a local crop seed supplier. At the time of the study, the trees were three years old. Eucalyptus and carob trees were transplanted using 6-month-old seedlings, while moringa trees were sown directly in the field.

The physiological responses of the three tree species were studied in three different water management treatments: subsurface drip irrigation (DI), tank irrigation (TI), and unirrigated plants (NI). In the subsurface drip irrigation treatment, the plants were watered weekly with 8 L/plant/week, while in the tank irrigation treatment, the plants were watered monthly with an average amount of 30 L/plant/month.

The experiment was set up using a split-plot experimental design with two classification factors (irrigation and species) and three blocks. The irrigation factors were assigned to the whole plots, while the species were assigned to the subplots with eight trees per species.

### Physiological parameters 

All the physiological parameters were measured once for the unirrigated plants and twice for the irrigated plants, before and after irrigation. Before irrigation, the trees in the drip irrigation plots were subjected to a week of drought, while the plants in the tank irrigation treatment remained without irrigation for a month.

### Relative water content

Relative water content (RWC) was measured on three fully expanded leaves per tree, sampled from the same position from nine trees per species and per irrigation treatment. The collected leaves were placed in plastic bags and left in a cooler over the sampling period to prevent water loss. After sampling, each leaf was cut off at the base using a blade and weighed to obtain the fresh weight (FW), then placed in a Petri dish with a piece of paper imbibed with distilled water and kept at room temperature in the dark for 12 h. Later, the excess water was wiped from the leaves and they were then weighed to obtain the turgid weight (TW). Finally, the leaves were placed in an oven at 60 °C for 72 h and weighed to obtain the dry weight (DW). The RWC value for each leaf was determined as follows:

$$RWC = \frac{FW - DW}{{TW - DW}} \times 100$$

### Leaf water potential

Leaf water potential was measured using a model 1000 PMS pressure chamber (PMS, Corvallis, Oregon, USA) using pressurized oxygen. For each irrigation treatment, three twigs bearing leaves were selected per species. Samples were taken of east-facing, west-facing, and south-facing twigs from three trees. All samples were taken at the same plant height.

Measurements were taken twice a day. First, the pre-dawn water potential (PWP) was measured between 4.30 am and 6.00 am Moroccan time to determine the tree water potential at equilibrium with the soil water potential. Later, the midday water potential (MWP) was measured between 1 pm and 2.30 pm to ascertain the plant hydric status under the transpiratory demand of the atmosphere.

### Stomatal conductance (mmol/m^2^/s)

Stomatal conductance was measured using a Delta-T AP4 (Delta-T Devices, Cambridge, UK) porometer. The measurements were made on three fully exposed leaves per species and per irrigation treatment.

### Statistical analysis 

Means, standard deviations, and coefficients of variation were calculated per species and per irrigation treatment. A one-way or two-way analysis of variance was performed depending on the number of studied factors to compare the different treatments. When the residuals were not normally distributed, the ANOVA model was adjusted using the Box-Cox method with an optimal λ. Then, Tukey's Honest Significant Difference (HSD) comparison test (*p* < 0.05) was performed to discriminate between the different means.

Descriptive statistics, analysis of variance, post hoc analysis and graph generation were carried out using R Core Team software (2020)^33^, the Agricolae package of De Mendiburu (2021)^34^, Modern Applied Statistics with S of Venables and Ripley^35^, and the Tidyverse package, Wickham et al.^36^.

### Ethics approval

The authors declare that all the methods and experiments carried out on *Ceratonia siliqua*, *Eucalyptus camaldulensis*, and *Moringa oleifera* are in accordance with relevant institutional, national, and international guidelines and legislation. These three forest species are not listed as endangered species or species at risk of extinction according to the IUCN Policy statement and the Convention on the Trade in Endangered Species. The National Agency for Water and Forests of Morocco provided the Carob and Eucalyptus seedlings. These seedlings were produced following Moroccan seedling production quality standards before being planted at the reforestation site. On the other hand, *Moringa oleifera* seeds were bought from a local crop seed supplier.

## Results 

### Relative water content

The average relative water content (RWC) values per species and per irrigation treatment, before and after irrigation, are given in Table 1.

**Table 1:** RWC mean values ± standard deviation measured before and after irrigation and F-Values from a one-way ANOVA per species and per irrigation treatment.

Irrigation	Before irrigation			After irrigation		
	EC	CS	MO	EC	CS	MO
DI (%)	82.6 ± 3.4 A	82.3 ± 4.5 A	77.3 ± 3.2 B	84.2 ± 3.7 A	84.2 ± 3.0 A	76.6 ± 3.1 A
TI (%)	84.2 ± 2.2 A	75.6 ± 6.2 B	81.4 ± 2.1 A	86.4 ± 2.7 A	79.4 ± 5.6 B	78.5 ± 2.2 A
NI (%)	78.2 ± 6.4 A	79.7 ± 2.3 AB	74.2 ± 3.8 B	–	–	–
*p* value	0.075^NS^	0.032*	0.007**	NS	*	NS

***, **, * significant at 0.1%, 1%, and 5% respectively; NS, Non-significant.

The means not sharing a letter are significantly different at a 95% confidence level.

DI, Drip irrigation; TI, Tank irrigation; NI, Unirrigated plants; EC, *Eucalyptus camaldulensis*; CS, *Ceratonia siliqua*; MO, *Moringa oleifera.*

Before irrigation, the highest RWC values were observed in *Eucalyptus camaldulensis* (82.0%), followed by *Ceratonia siliqua* (79.5%) and finally *Moringa oleifera* (77.8%).

When comparing irrigation treatments, no significant differences were observed in the eucalyptus species. Contrastingly, in the carob trees, we found low significant differences (*p* = 0.032) between the different irrigation treatments, with the highest value recorded in drip irrigation (82.29% ± 4.49), followed by unirrigated plants (79.67% ± 2.33) and tank-irrigated seedlings (75.56% ± 6.23). Finally, significant differences (*p* = 0.007) between the three irrigation treatments were found in moringa trees, with an average of 81.35% in tank irrigation and nearly 75% in drip-irrigated and unirrigated plants (Table 1).

After irrigation, the RWC values were slightly higher in eucalyptus and carob trees, with the highest average observed in *Eucalyptus camaldulensis* (85.5%), followed by *Ceratonia siliqua* (82.2%), and finally *Moringa oleifera* (77.7%). When comparing the two irrigation treatments, the RWC was significantly (*p* = 0.035) different only in the case of carob trees, with an RWC of 84.16% in drip irrigation compared to 79.36% in tank irrigation (Table 1).

Finally, an ANOVA analysis was performed to see if the RWC values across the irrigation schemes were significant per species. Correspondingly, the average RWC values per irrigation and measurement period with their pair comparison groups are given in Table 2. This table shows that a significant difference was only apparent between the average values of the two measurement periods in eucalyptus (*p* = 0.049).

**Table 2:** RWC mean values ± standard deviation per species, irrigation status and irrigation treatment.

Irrigation	EC	CS	MO
BI	**83.4 ± 2.9% B**	**78.9 ± 6.3% A**	**79.0 ± 3.4% A**
DI (BI)	82.6 ± 3.4% b	82.3 ± 4.5% a	77.3 ± 3.2% a
TI (BI)	84.2 ± 2.2% ab	75.6 ± 6.2% b	81.4 ± 2.1% b
AI	**85.3 ± 3.4% A**	**81.8 ± 5.0% A**	**77.4 ± 2.9% A**
DI (AI)	84.2 ± 3.7% ab	84.2 ± 3.0% a	76.6 ± 3.1% ab
TI (AI)	86.4 ± 2.7% a	79.4 ± 5.6% ab	78.5 ± 2.2% b

The means not sharing a letter are significantly different at a 95% confidence level, uppercase letters are used to differentiate between irrigation status and lowercase letters are used to differentiate between irrigation treatments at a given status.

Significant values are in bold.

BI, Before irrigation; AI, After irrigation; DI, Drip irrigation; TI, Tank irrigation; EC, *Eucalyptus camaldulensis*; CS, *Ceratonia siliqua*; MO, *Moringa oleifera.*

### Leaf water potential 

**Figure 1:**
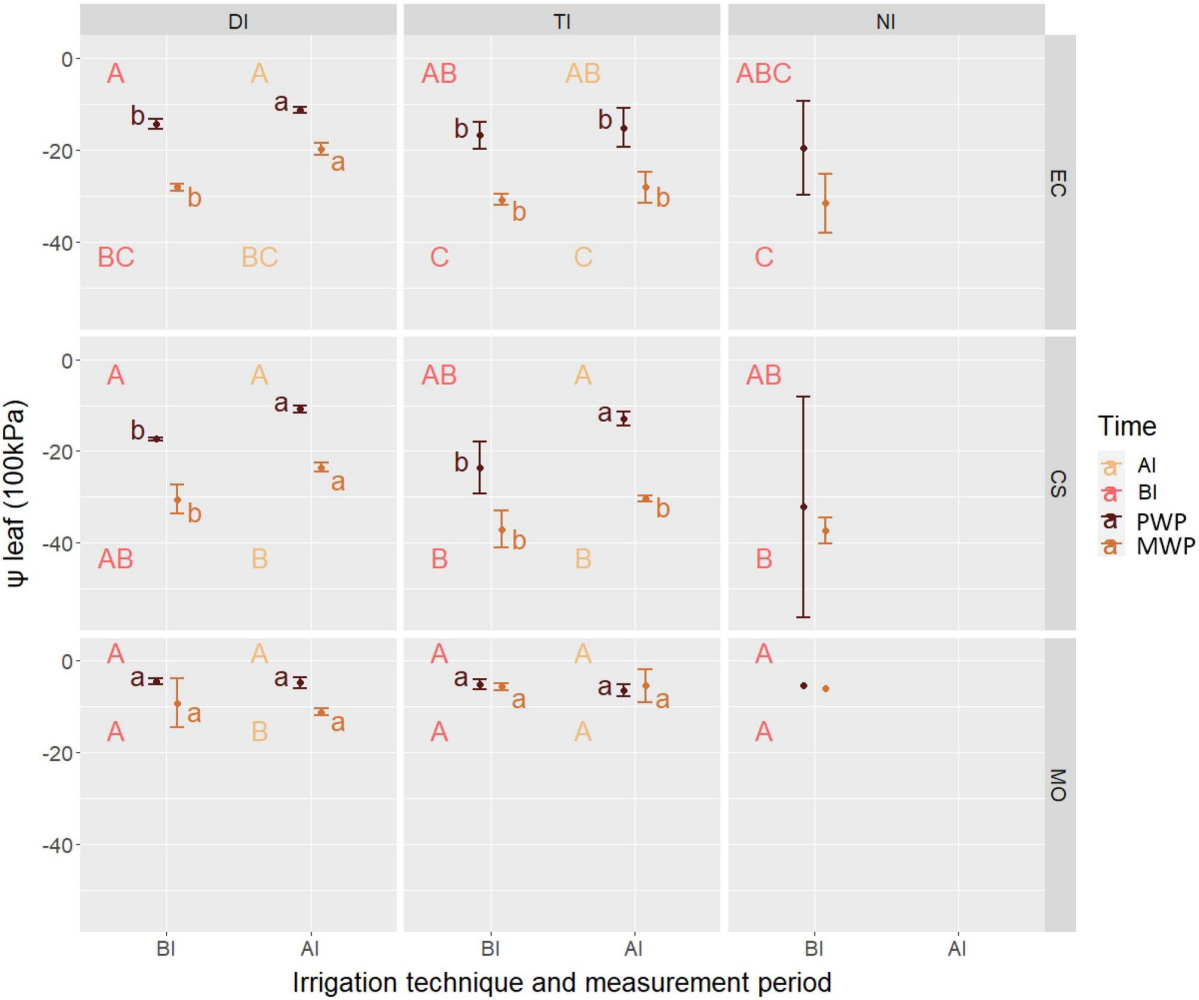
Mean ± standard deviation of PWP and MWP measured before (BI) and after (AI) irrigation per irrigation treatment and per species. HSD comparisons between Irrigation*Time interaction in a specific measurement period are indicated in uppercase, HSD comparisons between Irrigation*Period interaction in a specific time period are indicated in lowercase. DI: Drip irrigation; TI: Tank irrigation; NI: Unirrigated plants; EC: *Eucalyptus camaldulensis*; CS, *Ceratonia siliqua*; MO, *Moringa oleifera*; PWP, Pre-dawn water potential; MWP, Midday water potential; AI, After irrigation; BI, Before irrigation.

Before irrigation, the average recorded pre-dawn leaf water potential (PWP) was around − 16 bar in eucalyptus trees and − 24 bar in carob trees. In contrast, the average midday leaf water potential (MWP) values were − 30 and − 35 bar in eucalyptus and carob trees, respectively. For moringa trees, both PWP and MWP values remained around − 5 bar (Fig. 1).

The analysis of variance showed significant differences (*p* < 0.0005) between species for both PWP and MWP. While eucalyptus and carob means were found to be similar, they differed from the moringa values.

When comparing irrigation treatments, no significant difference was observed between the three treatments for PWP and MWP for all the species. However, when comparing pre-dawn and midday values, we found significant differences between the PWP and MWP values for eucalyptus (*p* < 0.0005) and carob trees (*p* = 0.002), whereas the difference was not significant in moringa. However, the interaction of the two factors (I*T) was not significant in any of the species (Table 3).

**Table 3:** Two-way ANOVA F-values and significance for Leaf Water Potential of differences between irrigation treatments, measurement times and their interaction per species and per irrigation period.

Period	Before Irrigation				After Irrigation			
Source	DF	EC	CS	MO	DF	EC	CS	MO
Irrigation (I)	2	2.13^NS^	0.08^NS^	12.74^NS^	1	12.74**	101.07***	5.98*
Time (T)	1	15.73***	0.53**	48.12^NS^	1	48.12***	803.9***	11.95**
I*T	2	0.39^NS^	0.19^NS^	0.21^NS^	1	0.21^NS^	56.68***	14.58**

DF: degrees of freedom, ***, **, * significant at 0.1%, 1%, and 5% respectively; NS, Non-significant.

EC, *Eucalyptus camaldulensis*; CS, *Ceratonia siliqua*; MO, *Moringa oleifera.*

After irrigation, no significant differences were observed between the species before and after irrigation for both PWP and MWP.

In contrast, the differences between irrigation treatments and measurement time (PWP and MWP) were significant for all species. However, the interactions of those factors were only significant for carob and moringa trees (Table 3). For eucalyptus trees, in drip irrigation, the average PWP and MWP values were − 11.33 bar and − 19.83 bar, respectively, while in tank irrigation, they were − 15.17 bar for PWP and − 28.17 bar for MWP (Fig. 1). For carob plants, the average PWP value for both irrigated treatments was around − 11 bar, while the mean MWP value was − 23.5 bar and − 30.33 bar in drip irrigation and tank irrigation, respectively (Fig. 1). Finally, in moringa plants, the average PWP value was around − 5 bar in both irrigation treatments. In contrast, the average MWP value was − 11.17 bar and − 5.5 bar in drip irrigation and tank irrigation, respectively (Fig. 1).

An ANOVA was performed to compare the values before and after irrigation. We found that irrigation significantly affected PWP (*p* = 0.011) and MWP (*p* = 0.001) in *E. camaldulensis*. Specifically, per irrigation treatment, both the PWP and MWP averages were statistically different (*p* < 0.05) before and after irrigation in the drip irrigation treatment, but in tank irrigation the means were similar (Fig. 1).

For *C. siliqua*, the results were the same as for the eucalyptus trees, where irrigation significantly affected PWP (*p* < 0.0005) and MWP (*p* = 0.001). Specifically, per irrigation treatment, PWP was significantly (*p* < 0.05) higher after irrigation in the drip and tank irrigation treatments. However, the MWP averages before and after irrigation were only different for the drip irrigation treatment (Fig. 1). Finally, in *M. oleifera*, irrigating did not affect leaf water potential measurements (Fig. 1).

### Stomatal conductance 

The mean values and standard deviations for stomatal conductance measured in the morning before and after irrigation for the different species and irrigation treatments are given in Table 4.

**Table 4:** Stomatal conductance mean values (mmol/m^2^/s) ± standard deviation per species, measurement period, and irrigation treatment.

	DI	TI	NI
EC			
BI	34.77 ± 5.32 A, b	24.87 ± 3.70 A, b	25.27 ± 2.73 A
AI	85.33 ± 13.32 a	33.17 ± 6.25 b	–
CS			
BI	73.00 ± 3.61 A, b	30.00 ± 10.97 C, c	58.33 ± 6.66 B
AI	212.00 ± 24.27 a	59.50 ± 11.30 b	–
MO			
BI	122.67 ± 12.34 A, b	116.33 ± 20.01 AB, b	85.00 ± 11.36 B
AI	319.33 ± 27.23 a	129.00 ± 34.00 b	–

The means not sharing a letter are significantly different at a 95% confidence level, uppercase letters are used to differentiate between irrigation status before irrigation, and lowercase letters are used to differentiate between means from different irrigation treatments and irrigation periods.

DI, Drip irrigation; TI, Tank irrigation; NI, Unirrigated plants; EC, *Eucalyptus camaldulensis*; CS, *Ceratonia siliqua*; MO, *Moringa oleifera*; BI, Before irrigation; AI, After irrigation.

Before irrigation, the stomatal conductance of *E. camaldulensis* did not differ significantly between the three irrigation treatments, remaining at around 25 mmol/m^2^/s (Table 4). However, for *C. siliqua,* stomatal conductance was significantly different between irrigation treatments (*p* = 0.001), with average values of 73 mmol/m^2^/s in drip-irrigated plants, 30 mmol/m^2^/s in tank-irrigated plants, and 58.33 mmol/m^2^/s in unirrigated plants (Table 4). In *M. oleifera*, stomatal conductance was weakly affected by the irrigation treatments (*p* = 0.042), while it was higher in the irrigated treatments than in the unirrigated plants. The average values were 122.67 mmol/m^2^/s, 116.33 mmol/m^2^/s, and 85 mmol/m^2^/s in the DI, TI and NI treatments, respectively (Table 4).

**Figure 2:**
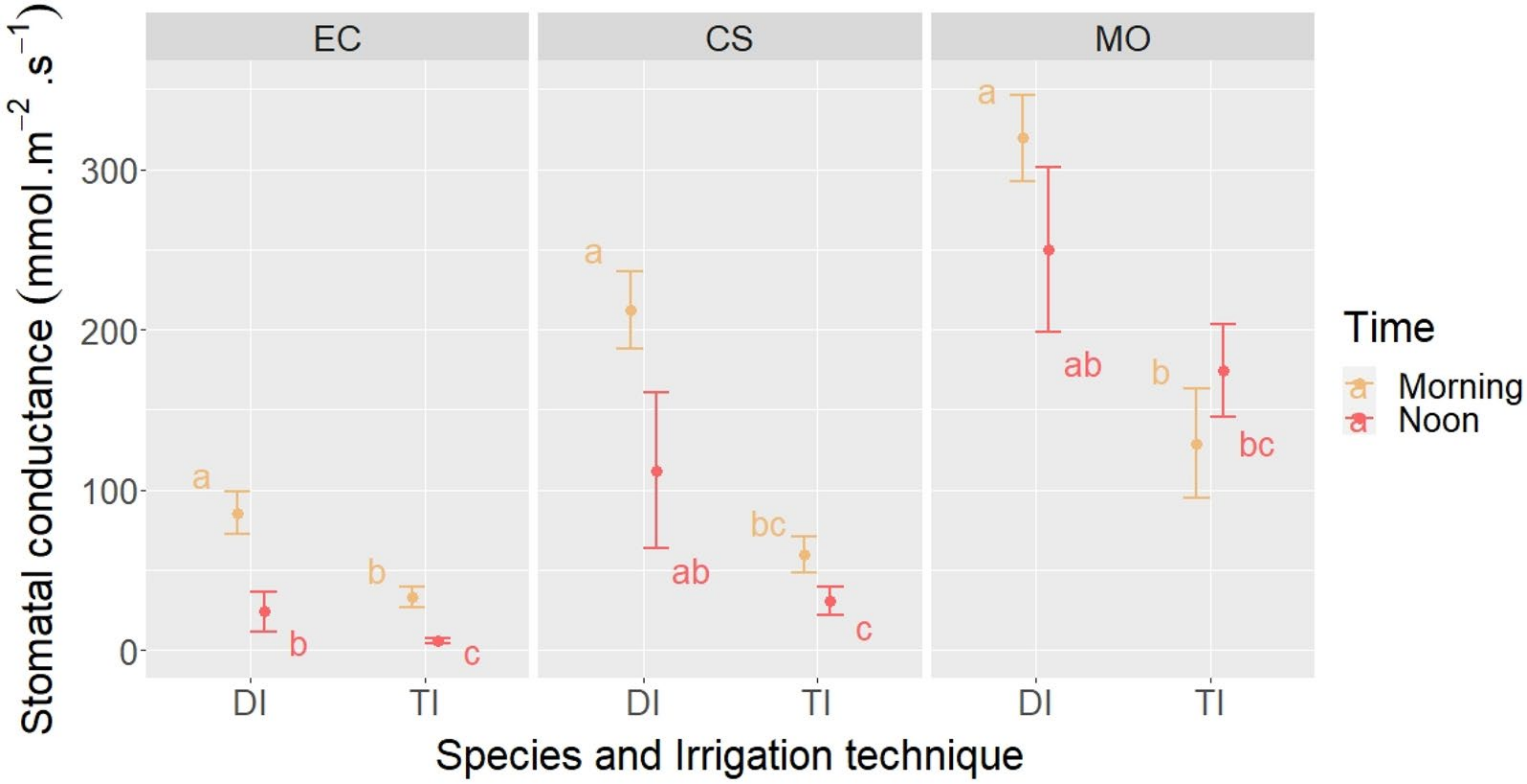
Mean ± standard deviation and Tukey's groups of morning and noon stomatal conductance after irrigation per species in drip and tank irrigation. EC, *Eucalyptus camaldulensis*; CS, *Ceratonia siliqua*; MO, *Moringa oleifera*; DI, Drip irrigation; TI, Tank irrigation.

After irrigation, the stomatal conductance of *E. camaldulensis* measured in the morning and at noon was strongly affected by the irrigation treatments (*p* < 0.001). There was also a very highly significant difference between the two measurement periods (*p* < 0.001). In drip irrigation, the stomatal conductance measured in the morning was 85.33 ± 13.33 mmol/m^2^/s, and it decreased to 23.87 ± 12.69 mmol/m^2^/s at noon, while in tank irrigation, it was 33.17 ± 6.25 mmol/m^2^/s in the morning and 5.55 ± 1.46 mmol/m^2^/s at noon (Fig. 2).

In *C. siliqua* plants, the irrigation treatments also significantly affected stomatal conductance (*p* < 0.001). In addition, we found a significant difference between the two measurement periods for the two irrigation treatments (*p* < 0.05). However, there was no significant difference between the measurement periods within each irrigation treatment. The stomatal conductance measured after irrigation was around 126 mmol/m^2^/s in drip irrigation and 44 mmol/m^2^/s in tank irrigation (Fig. 2).

Similarly, the stomatal conductance of *Moringa oleifera* plants was greatly affected by the irrigation treatments (*p* < 0.001). At the same time, we did not find any significant difference between the measurement periods for both irrigation treatments. The highest value was recorded in drip irrigation, with an average of 284 mmol/m^2^/s, compared to an average conductivity of 151 mmol/m^2^/s in tank irrigation (Fig. 2).

The stomatal conductance of *E. camaldulensis* plants measured in the morning before and after irrigation showed a significant difference between the two measurement periods in drip irrigation, with the highest value recorded after irrigation. Tank-irrigated plants showed no significant difference between the two measurement periods (Table 4). A similar trend was observed in moringa plants (Table 4). In carob plants, we recorded a significant difference between the two measurement periods in both irrigation treatments, with the highest values being observed after irrigation (Table 4). Finally, moringa trees followed the same behavior as eucalyptus, with higher values after irrigation in the drip-irrigated treatment, while the values were similar before and after irrigation in the tank-irrigated treatment.

## Discussion

Drought stress is a critical factor affecting plant growth and development in arid and semi-arid areas^37^^.^ This study looked at the physiological responses of three species (*C. siliqua*, *E. camaldulensis,* and *M. oleifera*) to water deficits in three irrigation regimes.

Drought impact and vulnerability assessments include the evaluation of the moisture degree in plants. For instance, leaf relative water content would be an important physiological indicator to assess water status in plants^38^. Leaf water potential has also proven to be a helpful stress index in several species^39^.

In a stressed state, the RWC of *C. siliqua* showed a high value in drip irrigation and unirrigated plants compared to tank irrigation. The stomata also remained open to a high degree in drip irrigation, followed by unirrigated plants, and to a lower degree in tank irrigation. PWP and MWP displayed low values reaching − 35 bar for MWP and − 20 bar for PWP in the three irrigation treatments. An efficient xylem transport system, or a deep root system that enhances the plant's ability to take up water, might help the plant maintain open stomata and a high relative water content under water deficit conditions. Our findings are consistent with the results obtained by Logullo & Salleo (1988) and Sakcalia & Ozturkb (2004)^21,40^. They demonstrated that carob trees possessing the widest xylem conduits are able to maintain open stomata and a high RWC under drought conditions in the field. In contrast, other studies reported that carob trees switched to a water-saving strategy under water stress with complete stomatal closure^22,41^. These contrasting results might be attributed to the seed's climatic origin, leading to seedlings with different genetic backgrounds and different physiological behaviors within this species. A previous report showed that *C. siliqua* seedlings from different provenances exhibited differing drought responses^42^. Likewise, Correia et al.^40^ reported that three carob cultivars behaved differently under water deficit conditions. Maintaining both open stomata and a high RWC at low leaf water potentials in this species is mainly associated with osmotic adjustment^43^. This could be considered as a strategy to tolerate water stress. Gadoum et al.^23^ reported that the accumulation of proline and soluble sugar in pot-grown carob plants under drought conditions constitutes a tolerance strategy that allows the maintenance of cell turgor at the highest possible level.

After rewatering, RWC, MWP, and stomatal conductance were higher in drip irrigation than in tank irrigation. In contrast, both irrigation treatments displayed the same value for PWP. Comparing these values to those obtained before rewatering showed no significant difference for RWC in both irrigation treatments. This could be attributed to increased stomatal conductance leading to water loss. Also, no significant difference was found for MWP in tank irrigation. Overall, the carob plants appeared more stressed in tank irrigation; even after rewatering, they maintained low MWP, RWC, and stomatal conductance values. It seems that the period between two irrigations (one month) did not allow the rapid recovery of the plant in this species. El Hafid et al.^44^ highlighted that the ability to recover upon rewatering depends on the species and the severity of the water stress imposed.

*E. camaldulensis* plants exhibited no difference in RWC before irrigation in all three irrigation treatments, which remained at around 80%. PWP and MWP were relatively low in the different irrigation treatments. We also found very low stomatal conductance in the morning. These results suggest that *E. camaldulensis* avoided drought by stomatal closure. It therefore appears that this species is highly water-saving under drought conditions. Similar results were reported in several studies^25,26,45^. As Bennet et al. (1987)^46^ demonstrated, the decrease in stomatal conductance under water deficit conditions allowed RWC to be maintained at high values. However, Merchant et al.^28^ showed that osmotic adjustment is a common response to water deficits in six Eucalyptus species (*E. camaldulensis*, *E. obliqua*, *E. rubida*, *E. cladocalyx*, *E. polyanthemos,* and *E. tricarpa*). In the unirrigated treatment, the plants were subjected to a long drought spell; however, they behaved similarly to irrigated plants, indicating that they can extract water from deep soil layers via an efficient root system. A descriptive study of the *E. camaldulensis* root system showed that the long central tap root and the numerous upper roots permitted access to large amounts of water stored in deep soil layers^47,48^. In this regard, Zahid et al.^49^ found that increased water uptake by the eucalyptus root system may reduce the aquifer resources for irrigated agriculture in arid lands.

After irrigation, stomatal conductance showed low values in tank irrigation and even stomatal closure at the warmest time of day (noon). Also, PWP, RWC, and MWP showed similar values to those obtained before irrigation. Although increased in plants watered by drip irrigation with low stomatal conductance at noon, RWC revealed similar values to those before irrigation. This might be due to the high stomatal conductance recorded in the morning in this irrigation treatment. Similarly, Eucalyptus plants could not fully recover after rewatering in tank irrigation.

Finally, *Moringa oleifera* plants showed different behavior from the other two species. Before irrigation, RWC displayed high values in the different irrigation treatments. The leaf water potential (PWP and MWP) did not differ significantly between the three irrigation treatments, remaining at − 5 bar. Neither was there any significant change in stomatal conductance. Consequently, the severity of water stress did not cause a severe response in moringa plants, since the stomata remained open at extreme water restriction levels. Similar results for stomatal conductance were reported by Vasconcelos et al.^30^ in a greenhouse experiment, where *M. oleifera* seedlings were subjected to water restriction for 21 days in four treatments (40, 60, 80, and 100% field capacity). *M. oleifera* has a swollen tuberous main root with very sparse lateral roots^50^, allowing water and nutrients to be taken up and stored. This species therefore maintained a high relative water content, high leaf water potential, and high stomatal conductance under severe water stress. Furthermore, *M. oleifera* is a medicinal plant, rich in amino acids, vitamins, and minerals, with a high economic interest in rural communities^51^. The concentration of these compounds increases under water stress, as reported in previous studies^31,32,52^, and their accumulation could enhance water uptake by increasing cell osmolarity. This species, also called the drumstick tree, is highly drought-tolerant and is cultivated in semi-arid and arid regions of India, Pakistan, Afghanistan, Saudi Arabia and East Africa, receiving an annual rainfall as low as 300 mm^29,53^.

After rewatering, all the evaluated parameters revealed no significant difference between the two irrigation treatments, except for stomatal conductance, which displayed very high values in drip irrigation. Similarly, there was no significant difference between the two measurement periods (before and after irrigation). Hence, this species did not show a strong physiological response using these parameters. *M. oleifera* is a deciduous species displaying a different acclimation mechanism by shedding leaves in response to water stress and limiting transpiration. Moreover, it has a tuberous root system with water and energy storage that allows the plant to grow new leaves when climatic conditions become favorable.

The three species studied showed three contrasting responses to water deficits: *Ceratonia siliqua* tolerated a water deficit by maintaining open stomata and a high leaf relative water content at a low water potential associated with osmotic adjustment. Additionally, the species might possess a deep root system enhancing water uptake. On the other hand, *Eucalyptus camaldulensis* avoids drought by closing its stomata, leading to the maintenance of a high leaf relative water content. Finally, *Moringa oleifera* showed high stomatal conductance, high water potential and a high relative water content compared to the other species. However, the species relies on water and energy storage in its root system and a leaf-shedding mechanism.

These physiological responses to water stress provide insights on how the species may survive in extreme drought conditions. Hence, the three studied species can be considered as suitable candidates for rehabilitating degraded semi-arid areas. Eucalyptus and carob trees were shown to be more adapted to areas with long drought periods and Moringa with more extended humid periods for energy storage.

## Conclusion

This study assessed the physiological behavior of three field-grown species in response to water deficits in three irrigation treatments, by assessing certain physiological parameters. The results revealed three contrasting physiological behaviors. First, *C. siliqua,* an evergreen indigenous species adapted to the Mediterranean climate, tolerates a water deficit by decreasing its leaf water potential and probably enhancing its root system depth. Second, *E. camaldulensis*, an evergreen species planted in Morocco, showed an isohydric behavior that resulted in stomatal closure under water deficit conditions. Finally, *M. oleifera* is an exotic deciduous species that responds to water deficits by losing its leaves. The physiological regulatory pathways help the plant to cope with water deficit stress, and hence allow species choice for adaptative environments. Thus, further investigations on the transcriptomic and metabolomic mechanisms involved in these species’ adaptation to drought are recommended.

## Data Availability

All data generated or analyzed during this study are included within the article.
